# Improving the Risk Assessment of Pesticides through the Integration of Human Biomonitoring and Food Monitoring Data: A Case Study for Chlorpyrifos

**DOI:** 10.3390/toxics10060313

**Published:** 2022-06-09

**Authors:** Jose V. Tarazona, Maria del Carmen González-Caballero, Mercedes de Alba-Gonzalez, Susana Pedraza-Diaz, Ana Cañas, Noelia Dominguez-Morueco, Marta Esteban-López, Irene Cattaneo, Andromachi Katsonouri, Konstantinos C. Makris, Thorhallur I. Halldorsson, Kristin Olafsdottir, Jan-Paul Zock, Jonatan Dias, Annelies De Decker, Bert Morrens, Tamar Berman, Zohar Barnett-Itzhaki, Christian Lindh, Liese Gilles, Eva Govarts, Greet Schoeters, Till Weber, Marike Kolossa-Gehring, Tiina Santonen, Argelia Castaño

**Affiliations:** 1 National Centre for Environmental Health, Instituto de Salud Carlos III, 28220 Madrid, Spain; mcgonzalez@isciii.es (M.d.C.G.-C.); malba@isciii.es (M.d.A.-G.); spedraza@isciii.es (S.P.-D.); acanas@isciii.es (A.C.); ndominguez@isciii.es (N.D.-M.); m.esteban@isciii.es (M.E.-L.); 2 European Food Safety Authority (EFSA), I-43126 Parma, Italy; irene.cattaneo@ext.efsa.europa.eu; 3 Cyprus State General Laboratory, Ministry of Health, Nicosia 1451, Cyprus; akatsonouri@sgl.moh.gov.cy; 4 Cyprus International Institute for Environmental and Public Health, Cyprus University of Technology, Limassol 3036, Cyprus; konstantinos.makris@cut.ac.cy; 5 Faculty of Food Science and Nutrition, School of Health Sciences, University of Iceland, 102 Reykjavik, Iceland; tih@hi.is; 6 Department of Epidemiology Research, Statens Serum Institut, DK-2300 Copenhagen, Denmark; 7 Department of Pharmacology and Toxicology, University of Iceland, 107 Reykjavik, Iceland; stinaola@hi.is; 8 National Institute for Public Health and the Environment (RIVM), Bilthoven, 3720 BA De Bilt, The Netherlands; jan-paul.zock@rivm.nl; 9 Wageningen Food Safety Research (WFSR), 6700 AE Wageningen, The Netherlands; jonatan.dias@wur.nl; 10 APB Provinciaal Instituut voor Hygiëne, 2000 Antwerpen, Belgium; annelies.dedecker@provincieantwerpen.be; 11 Department of Sociology, University of Antwerp, 2020 Antwerpen, Belgium; bert.morrens@uantwerpen.be; 12 Ministry of Health, Jerusalem 9446724, Israel; tamar.berman@moh.gov.il (T.B.); zoharba@ruppin.ac.il (Z.B.-I.); 13 Ruppin Research Group in Environmental and Social Sustainability, Ruppin Academic Center, Emek Hefer 4025000, Israel; 14 Division of Occupational and Environmental Medicine, Institute of Laboratory Medicine, Lund University, 22363 Lund, Sweden; christian.lindh@med.lu.se; 15 VITO Health, Flemish Institute for Technological Research (VITO), 2400 Mol, Belgium; liese.gilles@vito.be (L.G.); eva.govarts@vito.be (E.G.); greet.schoeters@vito.be (G.S.); 16 Department of Biomedical Sciences, University of Antwerp, 2020 Antwerp, Belgium; 17 German Environment Agency (UBA), 14195 Berlin, Germany; till.weber@uba.de (T.W.); marike.kolossa@uba.de (M.K.-G.); 18 Finnish Institute of Occupational Health, P.O. Box 40 Helsinki, Finland; tiina.santonen@ttl.fi

**Keywords:** human biomonitoring, chlorpyrifos, pesticide exposure, pesticide risk assessment, HBM4EU

## Abstract

The risk assessment of pesticide residues in food is a key priority in the area of food safety. Most jurisdictions have implemented pre-marketing authorization processes, which are supported by prospective risk assessments. These prospective assessments estimate the expected residue levels in food combining results from residue trials, resembling the pesticide use patterns, with food consumption patterns, according to internationally agreed procedures. In addition, jurisdictions such as the European Union (EU) have implemented large monitoring programs, measuring actual pesticide residue levels in food, and are supporting large-scale human biomonitoring programs for confirming the actual exposure levels and potential risk for consumers. The organophosphate insecticide chlorpyrifos offers an interesting case study, as in the last decade, its acceptable daily intake (ADI) has been reduced several times following risk assessments by the European Food Safety Authority (EFSA). This process has been linked to significant reductions in the use authorized in the EU, reducing consumers’ exposure progressively, until the final ban in 2020, accompanied by setting all EU maximum residue levels (MRL) in food at the default value of 0.01 mg/kg. We present a comparison of estimates of the consumer’s internal exposure to chlorpyrifos based on the urinary marker 3,5,6-trichloro-2-pyridinol (TCPy), using two sources of monitoring data: monitoring of the food chain from the EU program and biomonitoring of European citizens from the HB4EU project, supported by a literature search. Both methods confirmed a drastic reduction in exposure levels from 2016 onwards. The margin of exposure approach is then used for conducting retrospective risk assessments at different time points, considering the evolution of our understanding of chlorpyrifos toxicity, as well as of exposure levels in EU consumers following the regulatory decisions. Concerns are presented using a color code, and have been identified for almost all studies, particularly for the highest exposed group, but at different levels, reaching the maximum level, red code, for children in Cyprus and Israel. The assessment uncertainties are highlighted and integrated in the identification of levels of concern.

## 1. Introduction

In most jurisdictions around the globe, pesticides are subjected to specific authorization requirements [1]. The regulatory decisions are supported by scientifically-based pre-marketing assessments and re-assessments, complemented by enforcement actions including monitoring programs. The authorization of use patterns, also named good agricultural practice (GAP), is supported by setting maximum residue levels in food commodities, established by the Codex Alimentarius at international level as well as by specific legislation within each jurisdiction, such as Regulation (EC) No 396/2005 in the EU.

The risk assessment process follows the standard paradigm for chemical risk assessment. The hazard assessment identifies possible hazards and establishes health-based guidance values (HBGV): the acceptable daily intake (ADI) for chronic life-long exposures, the acute reference dose (ARfD) for exposures within a single day or single meal, and the acceptable operator exposure level (AOEL) for non-oral exposures. The dietary exposure assessment combines the expected or measured pesticide residue levels in food commodities with the consumption of each commodity according to standard diets. While efforts for developing methodologies for combining the risk of concurrent exposure to different pesticides are ongoing, most current regulatory decisions are still based on a substance-by-substance approach.

While these prospective risk assessments are essential for preventing consumer exposure to levels of concern, they are based on agreed assumptions and not on actual consumer exposure. Human biomonitoring (HBM) of relevant markers complements the information with retrospective assessments, and is emerging as a key tool for calibrating and validating the regulatory risk assessment models. The European Human Biomonitoring Initiative (HBM4EU, www.hbm4eu.eu, accessed on 4 May 2022) is a European Joint Program, which aims to harmonize and use biomonitoring to understand human exposure to chemicals in the environment, in occupational settings or in non-occupational settings through the use of consumer products, and the related health risks, in order to improve chemical risk management and to support policy making [2]. In the frame of HBM4EU, aligned biomonitoring studies from across Europe [3,4] provided harmonized internal exposure data to specific chemical substances in different age groups of the general population. Studies included in HBM4EU-aligned studies met a set of inclusion criteria, such as minimal number of participants, minimal set of variables, specific time period for data collection, laboratory QA/QC, as well as specific conditions for reporting. These criteria are not always explicitly considered in published monitoring studies. In order to further explore the comparison of both monitoring approaches, this work includes a second set of human biomonitoring studies, retrieved from a scientific literature search. As chlorpyrifos has been extensively studied, it was decided to focus on a single EU country, not included in the selected aligned studies, and with a number of available monitoring studies covering at least a decade.

It is particularly interesting to compare the risk estimates based on the occurrence of the substance in food with those based on biomonitoring in humans, as both represent real exposure levels; and the organophosphate insecticide chlorpyrifos offers a stimulating case study. In the last decade, the initial acceptable daily intake (ADI), established in 2006 as 0.01 mg/kg body weight per day, has been reduced several times following EFSA assessments, triggering a reduction in the authorized use and maximum residue levels (MRLs) and, therefore, a progressive reduction in the estimated exposure of European citizens. In 2012, a review was required due to new toxicological studies, and two years later the EFSA proposed a reduction of the ADI to 0.001 mg/kg body weight per day [5]. The EFSA assessment triggered a re-assessment of the MRLs in 2015 [6], which were incorporated into the EU legislation in 2016. In 2019, the EFSA concluded that toxicological reference values for this substance could not be established [7]. Similar conclusions were obtained for chlorpyrifos-methyl [8]. Subsequently, in 2020, the approval of the active substance chlorpyrifos was not renewed and existing authorizations for plant protection products containing chlorpyrifos in the EU Member States were revoked; and all MRL were set at the default value of 0.01 mg/kg as indicated in the EU legislation.

Chlorpyrifos exposure can be monitored in humans through two urine biomarkers, 3,5,6-trichloro-2-pyridinol (TCPy) and the group of alkyl phosphates (AP). Biological reference values for both biomarkers have been proposed [9]. TCPy is a common metabolite with the closely related pesticide chlorpyrifos-methyl and has been selected for the work presented herein. The previously proposed reference values should be updated considering the recently raised concerns on genotoxicity potential and developmental neurotoxicity. Following the EFSA assessment that an ADI can no longer be proposed, the risk assessment presented here is based on the alternative approach, quantifying the margins of exposure for a set of selected points of departure (PoD).

## 2. Materials and Methods

### 2.1. Data and Information Sources

Toxicity data were retrieved from EFSA conclusions and related documents published in the EFSA Journal https://efsa.onlinelibrary.wiley.com/journal/18314732 (accessed on 25 March 2022) or publicly available through the Open-EFSA web https://open.efsa.europa.eu/ (accessed on 25 March 2022). A literature search using Web Of Science and SCOPUS was conducted for retrieving toxicokinetic information focusing on studies with human volunteers.

Aggregated (percentiles for the full dataset) HBM data (TCPy) were obtained from HBM4EU-aligned studies measuring this metabolite of chlorpyrifos. These included six studies on adults (PT INSEF, Study CH, IL RAVMABAT, FR ESTEBAN, IS Diet_HBM, DE ESB), with an age range of 20–39 years, from France, Germany, Iceland, Israel, Portugal and Switzerland, conducted in the period 2014–2021; and six studies on children (FR Esteban, SI SLOCRP, NL SPECIMEN, BE 3xG, CY Organiko, IL RAVMABAT), with an age range of 6–11 years, from Belgium, Cyprus, France, Israel, Slovenia and the Netherlands, conducted in the period 2014–2020.

A literature search, using Web Of Science and SCOPUS, was conducted for retrieving published monitoring data. Following a pre-screening, Spain was selected as a relevant country for covering published human biomonitoring studies from projects other than HBM4EU, providing a complementary assessment for children and adult exposure in Southern Europe. The search focused on data from Spain and the list of studies was completed with a secondary search covering the references and citations of the identified studies.

### 2.2. Estimation of HBM Values

The HBMPoD represents the human biomonitoring PoD estimated through the adaptation of the general equation proposed under HBM4EU [10] for deriving human biomonitoring guidance values (HBM-GV). The HBGV is replaced by the PoD and combined with the molar urinary excretion fraction of the metabolite TCPy (Fue(TCPy)), corrected by the molecular weight, MW, differences. The adapted equation is:

(1) $$\mathrm{HBM}-\mathrm{PoD}\left(\mathrm{endpoint}\right)=\frac{\mathrm{PoD}\left(\mathrm{endpoint}\right)\;\times \;\left[\frac{\mathrm{MW}\left(\mathrm{TCPy}\right)\;\times \;\mathrm{Fue}\;\left(\mathrm{TCPy}\right)}{\mathrm{MW}\left(\mathrm{chlorpyrifos}\right)}\right]}{\mathrm{Daily}\;\mathrm{urinary}\;\mathrm{excretion}\;\mathrm{adjusted}\;\mathrm{to}\;\mathrm{the}\;\mathrm{bw}}$$

The hazard assessment is based on the EFSA conclusions published in 2011 [11], 2014 [5] and 2019 [7]. The reasoning for the evolution of the proposed ADI since 2011 and the most recent conclusion, are analyzed. Several points of departure—PoD (also named toxicological reference points—RP) have been selected to cover the most relevant observed effects. These PoDs represent the no-observed-adverse-effect level (NOAEL), lowest-observed-adverse-effect level (LOAEL) or benchmark dose (BMD) for specific effects extracted from the dose–response curve of the relevant studies. The uncertainties mentioned in the EFSA assessment [6] are considered for the interpretation of the margins of exposure (MoEs).

The toxicokinetic information was retrieved from a literature search (SCOPUS and Web of Science) focusing on human data.

### 2.3. Estimation of Urinary TCPy Levels from EU Food Monitoring Data

Urinary TCPy levels (µg/L) were calculated from the reported dietary risk data for different European diets (expressed in highest calculated exposure in % of ADI) retrieved from the EFSA Pesticide Residues Intake Model (PRIMo) [12] included in the European Union Annual Reports on pesticide residues in food from 2012 to 2019 [13,14,15,16,17,18,19,20]. Considering that TCPy is a common metabolite for chlorpyrifos and chlorpyrifos methyl, the external dose in mg/kg bw per day was estimated from the reported risk and the selected ADI value of both compounds. The obtained values were converted into molar units (mol/kg bw/day) and the molar contribution of TCPy from chlorpyrifos and chlorpyrifos methyl was summed up for each relevant diet. The total urinary TCPy concentration (µg/L) was then calculated, applying the TCPy molar excretion fraction of 0.7 [21] and assuming a urinary output of 24 mL/kg bw/day [22]. It should be noted that this estimation does not cover possible exposure from non-dietary sources.

### 2.4. Risk Characterization

Both prospective and retrospective risk assessments are based on the MoE approach; applied to each selected HBM-PoD endpoint to express the risk as the MoE(endpoint) according to the following equation:

(2) $$MoE\left(endpoint\right)=\frac{HBM-PoD\left(endpoint\right)}{Exposure\;level}$$

Prospective biomarker exposure estimations were calculated by applying toxicokinetic considerations to chlorpyrifos and chlorpyrifos-methyl exposure estimations, as published in EFSA Annual Reports on monitored levels in food.

Retrospective biomarker exposure estimations included data provided by the HBM4EU project and a literature search focused on published biomonitoring data for Spain (SCOPUS and Web of Science).

The risk characterisation was based on individualised comparisons of the observed MoEs with the relevant uncertainty factors, covering both generic (i.e., interspecies and intraspecies differences, non-threshold genotoxicity mechanisms) and specific uncertainties connected to the hazard, toxicokinetic and exposure data.

## 3. Results and Discussion

### 3.1. Evolution of Chlorpyrifos Hazard Characterisation in the EU

Chlorpyrifos was included in Annex I to Directive 91/414/EEC in 2006 by CD 2005/72/EC and the following reference doses were established: ADI and AOEL: 0.01 mg/kg bw per day, and ARfD: 0.1 mg/kg bw per day based on brain cholinesterase (AChE) inhibition and neurotoxic findings, respectively, as critical endpoints.

In 2013, after revision of new toxicological studies and scientific papers, the EFSA peer-review expert meeting agreed on the use of the red blood cell (RBC) AChE inhibition, instead of brain AChE inhibition, to derive the reference values, which were established as 0.001 mg/kg bw per day for both ADI and AOEL, and 0.005 mg/kg bw for ARfD [5].

After the process of renewal of approval, initiated in 2017, and the PPR 01 Experts’ meeting in 2019, experts agreed that the point of departure (PoD) for chlorpyrifos should be the developmental neurotoxicity (DNT) LOAEL of 0.3 mg/kg per day [7]. Regarding the general assessment, it was concluded that there were several uncertainties: the genotoxicity potential remains unclarified and the effects recorded in the DNT rat study indicate a concern that was supported by the epidemiological evidence related to developmental neurological outcomes in children for chlorpyrifos. In any case, overall, no reference values could be set because of the unclear genotoxicity potential of chlorpyrifos. Moreover, the approval of the active substance chlorpyrifos was not renewed and all existing authorizations for plant protection products containing chlorpyrifos in EU Member States were revoked by February 2020. The pesticide residues and MRLs (mg/kg) for chlorpyrifos were set at the default lowest limits of analytical determination (LODs) of 0.01 mg/kg in all products. The regulation was in force as of 13 November 2020.

In line with the more recent EFSA assessment [7], the following PoDs for chlorpyrifos were selected for this risk assessment:

- Overall PoD based on the DNT study on rats, as adverse effects were observed at the lowest tested dose the PoD was the LOAEL of 0.3 mg/kg bw per day
- Relevant long-term NOAEL 0.1 mg/kg bw per day, also applicable to parental toxicity and maternal NOAEL
- Short-term NOAEL for red blood cells AChE inhibition 0.1 mg/kg bw per day, same value as above but related to short-term exposures
- Relevant offspring NOAEL 1 mg/kg bw per day
- Relevant reproductive NOAEL 5 mg/kg bw per day
- Relevant carcinogenicity NOAEL 10 mg/kg bw per day (highest dose tested)

The specific toxicity of the metabolite TCPy, which is part of the residue definition in food, was also assessed and EFSA proposed an ADI of 0.06 mg/kg bw per day [5,6,7].

The EFSA assessment for chlorpyrifos-methyl [8] proposes the same overall PoD (based on chlorpyrifos study) and also for long-term toxicity. No value is proposed for short-term AChE, and the other PoDs are slightly higher: 3, 10 and 40 mg/kg bw per day for offspring, reproductive and carcinogenicity, respectively [8].

### 3.2. Proposed HBM-PoDs

The metabolism of chlorpyrifos has been extensively studied in both animals and humans [7,23]. Basically, the fraction of chlorpyrifos absorbed in the intestine is nearly completely converted to equimolar amounts of TCPy and alkyl phosphate metabolites; and both have been used as potential human biomarkers [24]. Alkyl phosphates cover a broad group of pesticides, while TCPy is only common in the closely related pesticide chlorpyrifos-methyl; thus TCPy was selected for this assessment. In a pharmacokinetic study with six human volunteers [21], the percentage of the administered oral dose recovered in urine as TCPy ranged between 49–81% with an average of 70%. The predicted percentage of absorbed dose in the same volunteers ranged between 52–84%, with an average of 72%. The good correlation between absorption and molar TCPy excretion confirms the capacity of TCPy as a biomarker with equimolar conversion of the absorbed dose. Other studies with human volunteers [25] and animals [5,7] have identified higher oral absorption rates, up to 93%. Based on these findings, the value of 70% was selected as the central estimate within a 50–93% range, corresponding to a molar urinary excretion fraction, Fue, of 0.7 (0.5–0.93 range). The molar ratio of TCPy to chlorpyrifos is 0.566. The 24 h daily urinary excretion selected by HBM4EU is 0.02 L/kg bw for adults and 0.03 L/kg bw for children [26].

Following this selection and the equation above, Table 1 presents the proposed HBM-PoDs to be used in the risk characterization.

In line with the EFSA assessment [8], the proposed HMB-PoDs are also relevant for exposure to chlorpyrifos-methyl and or a combination of both pesticides; being slightly conservative for the last three endpoints in the cases where chlorpyrifos-methyl is the main contributor.

### 3.3. Prospective Exposure Assessment Based on Monitored Levels in Food

**Figure 1:**
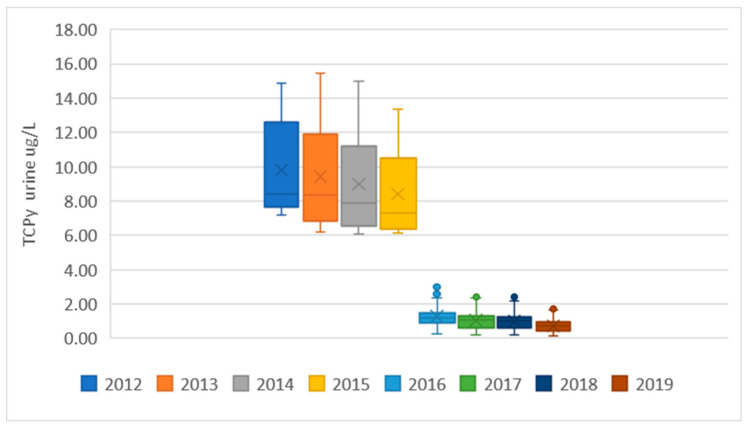
Predicted TCPy levels in EU consumers, estimated from chlorpyrifos levels in food extracted from the EU annual monitoring programs between 2012 and 2019. Data presented as box and whisker plots (see text for details).

The predicted TCPy urinary levels in the EU population, estimated from monitoring of chlorpyrifos in foodstuffs, are presented in Figure 1, using data from the European Union Annual Reports on pesticide residues in food from 2012 to 2019 [13,14,15,16,17,18,19,20]. Each EU report includes estimations for several diets; some diets cover generic clusters, while others are provided by the EU Member States for generic or specific national population groups, including both adults and children. Since 2012, the EFSA has updated PRIMo several times and the updates have included modifications in the diets in each version. In order to use exclusively publicly available data, the estimations were conducted for the diets reported for each year. The summary statistics are presented in Figure 1 as box and whisker plots presenting the range (vertical lines), upper and lower quartiles (box), if needed outliers covering the maximum and the minimum estimations (single dots), the average value (x) and the 50th percentile (horizontal line in the box).

The results showed a drastic reduction in exposure levels between 2015 and 2016, reflecting the regulatory measures adopted in the EU, as well as a general tendency towards exposure reduction between 2012 and 2015 and between 2016 and 2019. Individual estimates for each diet are reported in Table S1 at the Supplementary Material section.

### 3.4. Retrospective Exposure Assessment Based on Human Biomonitoring

#### 3.4.1. HBM4EU Data

Aggregated TCPy HBM data from HBM4EU-aligned studies measuring this biomarker for chlorpyrifos exposure in the general European population are presented in Table 2. These studies cover children from 6–11 years of age, in the period 2014–2020 and young adults 20–39 years of age, in the period 2014–2021 from different European countries and Israel. The 50th and 95th percentiles were selected for representing the exposure levels of the average population and the highest exposed group, respectively. In addition, the upper level of the 95th confidence interval of the 95th percentile has been selected for assessing the uncertainty in the exposure levels of the highest exposed group.

#### 3.4.2. Spanish Human Biomonitoring Data

**Figure 2:**
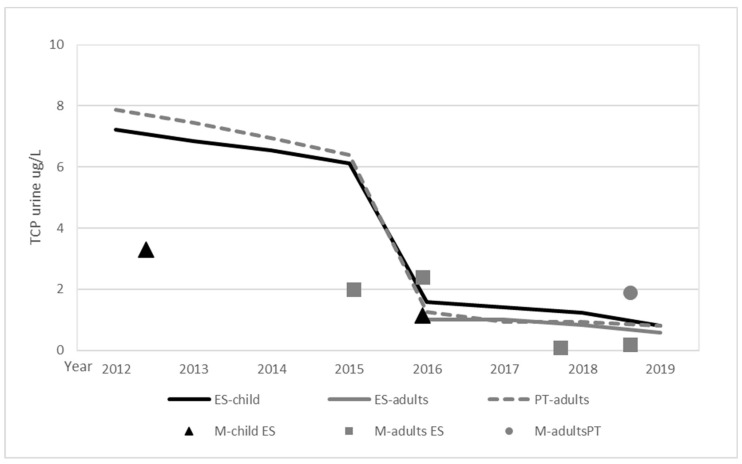
TCPy levels estimated from food (lines) vs. human monitoring (M-) for Spain and Portugal.

The available biomonitoring studies covering the Spanish population have been recently reviewed Yusa et al. [27]. No other studies were identified in the literature search. The studies cover children and several adult groups, and are distributed between 2003 and 2019. Setting comparisons is a challenge due to differences in both the study design and the reported information. Roca et al. [28] only reported creatinine adjusted values, the data were corrected to unadjusted values using the correlation derived from creatinine adjusted vs. non-adjusted data from Fernandez et al. [29]. Figure 2 summarizes the comparison of TCPy urinary levels estimated from food with those from human monitoring for the general population, including data for pregnant and lactating women while excluding farm workers. As the data for Spanish adults only covers the 2016–2019 period, data from the other Iberian country, Portugal, have been added to support the comparison.

Although the data available for the comparison are limited, the results suggest a general agreement between median measured levels and those estimated from food monitoring and the diets for Spain and Portugal. For five databases, information on both 50th and 95th percentiles was provided, the 95/50 ratio was around four for three databases and around 10 for the other two, confirming high individual variability.

### 3.5. Estimated MoEs for the Prospective Assessment

**Figure 3:**
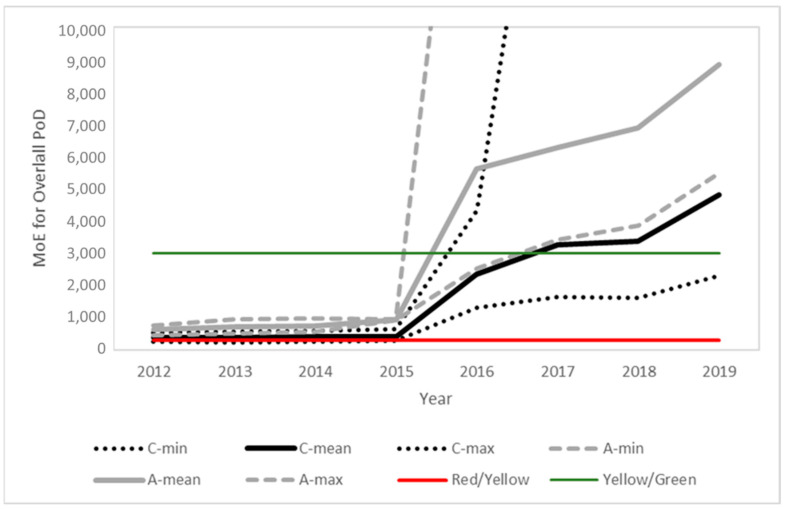
Time evolution of the margins of exposure for children (black) and adults (grey) for the overall PoD predicted from the food monitoring data. Solid lines represent the mean value and dotted lines the maximum and minimum values for children (C) and adults (A), respectively. Color lines indicate the thresholds between the risk levels (see Section 4 for details).

**Figure 4:**
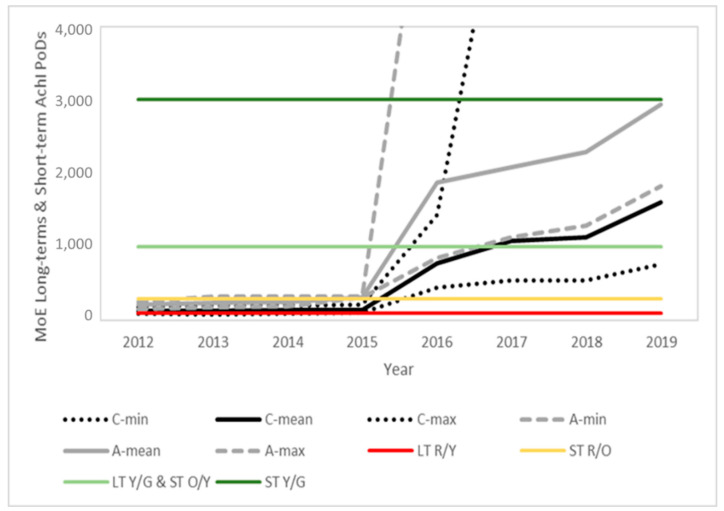
Time evolution of the margins of exposure for children (black) and adults (grey) for the long-term and short-term AChE PoDs predicted from the food monitoring data. Solid lines represent the mean value and dotted lines the maximum and minimum values for children (C) and adults (A), respectively. Color lines indicate the thresholds between the risk levels (see Section 4 for details).

**Figure 5:**
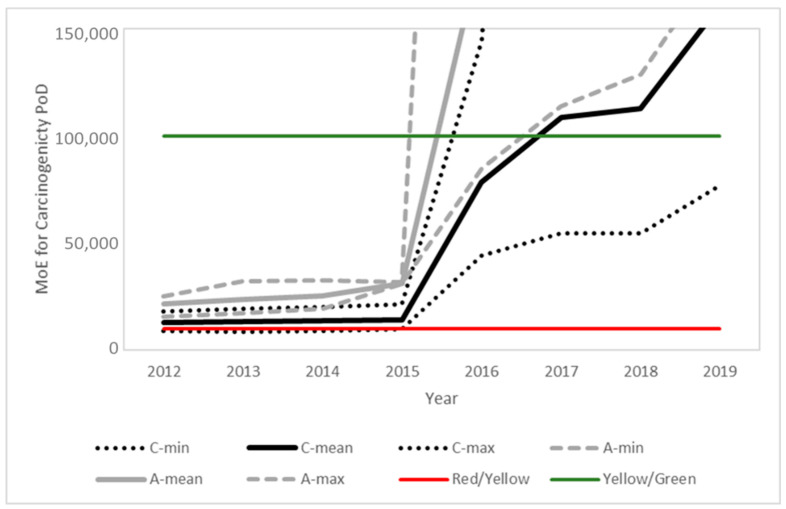
Time evolution of the margins of exposure for children (black) and adults (grey) for the carcinogenicity PoD predicted from the food monitoring data. Solid lines represent the mean value and dotted lines the maximum and minimum values for children (C) and adults (A), respectively. Color lines indicate the thresholds between the risk levels (see Section 4 for details).

Figure 3, Figure 4 and Figure 5 present the time evolution (from 2012 to 2019) of the estimated MoEs for the different EU diets and endpoints. European Union annual reports on pesticide residues in food from 2012 to 2019 included modifications in the diets used in different years. The comparison was based on the minimum, average and maximum estimations for the children and adult/general diets used each year. The proposed thresholds (see Section 4, Risk Characterization) have been included in the figures to facilitate the results’ interpretation.

In line with the exposure reduction, a clear temporal trend is observed for all PoDs, with a significant point of inflection in 2016, coinciding with the modification of the MRLs in this same year, following the 2014 EFSA conclusion and 2015 EFSA MRL review report. Further modifications in the MRLs and also regarding actual use contributed to additional temporal trends.

### 3.6. Estimated MoEs for the Retrospective Assessment

#### 3.6.1. HBM4EU Data

The estimated MoE ranges for the HBM4EU-aligned studies reporting TCPy urinary levels are summarized in Table 3. The MoEs have been estimated for the percentiles 50th; 95th and its upper 95th confidence interval, in order to characterize the risk for the average population, the high exposed group, and the highest exposed individuals, respectively. Individual estimations for each country and population group are reported in Table S2 in the Supplementary Material section.

#### 3.6.2. Spanish and Portuguese Populations

The estimated MoE ranges for the available Spanish studies reporting TCPy urinary levels and for the HBM4EU dataset from Portugal are summarized in Table 4. MoEs have been estimated for the 50th percentile (or the geomean if not available); 95th (or the 75th if not available), and the maximum value (or upper 95th confidence interval), in order to characterize the risk for the average population, the high exposed group, and the highest exposed individuals, respectively.

## 4. Risk Characterization

The interpretation of the MoEs required specific consideration, based on the selected PoD and the associated health concerns. Some were based on the extrapolation factors proposed for the derivation of HBGV [34,35,36], while others were specifically associated with the assessment of substances with potential genotoxicity concerns [37].

As a first step, any MoE below 100 for a PoD based on animal studies should be considered as a clear concern, considering the minimum factor of 100 required to cover intra- and interspecies extrapolation when setting HBGVs.

The overall PoD is based on a LOAEL instead of a NOAEL, requiring an additional factor. According to the ECHA guidance, a minimum factor of three is generally applicable, while a factor of up to 10 may be needed on some occasions. Consequently, any MoE below 300 represented a concern, and the concerns may be extended to MoEs between 300 and 1000, as the EFSA assessment [7] identified a number of relevant limitations for this study, including reduced exposure duration that would also trigger the need for an additional factor.

When the MoE was based on a short-term PoD, additional considerations were needed for assessing the monitoring data reflecting chronic exposure. An uncertainty factor (UF) of two is proposed by the EFSA and ECHA for the extrapolation of subchronic to chronic studies, while larger factors are needed for acute to subacute and subacute to subchronic extrapolations. The available study included acute (single dose) and subacute (repeated exposure from postnatal day 11 to 22), and a factor of five was identified between the acute and subacute NOAELs. For the subacute to subchronic extrapolation, ECHA recommends a factor of three, the extrapolation from subacute to chronic is not recommended by EFSA. Consequently a factor of three could be considered, however, it should be noted that according to the EFSA assessment [7], for chlorpyrifos the same NOAEL for RBC AChE activity inhibition of 0.1 mg/kg bw per day is applicable for both short-term and long-term exposure, thus the use of an additional factor of three is conservative in this specific case.

For the assessment of carcinogenicity, EFSA [35] proposed that an MoE of 10,000 or higher based on the benchmark dose level (BMLDL_10_) from an animal carcinogenicity study, and taking into account all uncertainties, could be considered of low concern for public health. Although a BMLDL_10_ was not available, the EFSA assessment concluded that for chlorpyrifos, carcinogenicity was of low concern and proposed a PoD based on the NOAEL that could be used as surrogate. This approach has been developed for impurities and other substances not intentionally added to food. As chlorpyrifos is no longer authorized in the EU, the approach is applicable for an assessment based on the current situation, although the monitoring data represented residues in line with the MRLs established at the time of monitoring.

One additional element is the consideration of the severity and nature of the observed effect. Specifically for pesticides the legislation indicates that an additional factor may be considered and applied for some effects including development neurotoxicity, and factors up to 10 have been applied in some pesticide risk assessments.

Based on these considerations, the MoEs can be grouped into four risk categories:

- RED: Confirmed concern: the MoE is lower than the requirements for uncertainty factors in standard assessments, i.e., lower than 100 for the NOAELs, lower than 300 for the LOAELs, and lower than 10,000 for the carcinogenicity of genotoxic substances
- ORANGE: Possible concern: the MoE is lower than the requirements for uncertainty factors in standard assessments plus the upper range regarding additional considerations for the extrapolation (factor of 10 for NOAEL to LOAEL, and factor of 3 for subacute to subchronic extrapolation)
- YELLOW: Concerns cannot be excluded. MoEs higher than those above but not offering an additional margin of 10.
- GREEN: Risk cannot be excluded due to the concerns on genotoxicity but it is expected to be very low: MOEs providing an additional margin of at least 10 from those of possible or confirmed concern.

According with this proposal, a summary of the risk characterization is graphically presented in Table 5.

The comparison of the published biomonitoring data for Spanish population groups with the HBM4EU-aligned studies indicated some common elements as well as relevant differences. Commonalities were observed regarding the diversity of the results but with a higher risk for children than for adults, and the high relevance of the overall and short-term PoD endpoints. The consistency is particularly relevant when comparing the Spanish data with the aligned HBM4EU data for Portugal, further supporting the similarities observed in the levels estimated from the food monitoring data for the two Iberian countries (Figure 3, Figure 4 and Figure 5). The highest risks were identified in a study conducted by Roca et al. [28] in 2014, before the 2016 modification of MRLs in the EU, and for Israel, not covered by the EU MRL regulation. Some Spanish datasets focused on a specific population groups. An interesting finding, to be further investigated, was the low risk observed for Spanish pregnant women, observed by Llop et al. [31] for samples taken in 2003–2006, when high MRLs were allowed in the EU, and confirmed by Bravo et al. [38] for samples collected in 2016–2017. The measured TCPy levels were similar or lower than those reported in other areas of the world [39,40,41], thus the risk assessment conclusions can be extrapolated to other regions.

The variability within the sampled population is evidenced by the change in color, and was large for some, but not all, databases.

## 5. Conclusions

Human biomonitoring data provide information on actual exposure levels, and in combination with guidance values, on risk levels including intrapopulation variability. The main limitation is the availability of proper urinary biomarkers; for chlorpyrifos TCPy also covers exposure to the closely related pesticide chlorpyrifos-methyl, as well as direct exposure to the metabolite itself, as TCPy is part of the residue definition in several food commodities. The proposal presented in this study demonstrates that this approach can be extended to those cases when an HBGV cannot be established. The combination of several PoDs, covering endpoints with different levels of concern for public health, and different exposure values, provide informative risk characterizations to support decision making. For chlorpyrifos, the monitoring data have confirmed the need for action, providing support to the regulatory decisions adopted in the EU. Promising results have been obtained regarding the comparison of the prospective assessment using monitoring data in food, and retrospective assessments using human biomonitoring; however additional studies are needed to generalize this opportunity; and this approach should be further explored using other pesticides in order to improve current predictive models through a calibration exercise. These improved models could be applied for pesticides with no suitable biomarkers for human biomonitoring, as well as for updating the premarketing risk assessments supporting the authorization and setting of maximum pesticide residue levels.

## Figures and Tables

**Table 1:** Proposed human biomonitoring points of departure (HBM-PoD) for chlorpyrifos following the EFSA [6] hazard characterization.

Endpoint	EFSA PoD Value mg/kg bw day	HBM-PoD Adults mg/L	HBM-PoD Children mg/L
Overall (based on DNT LOAEL)	0.3 ^1^	5.94	3.96
Long-term and maternal toxicity NOAEL	0.1	1.98	1.32
Short-term NOAEL for red blood cells AChE	0.1	1.98	1.32
Offspring NOAEL	1	19.81	13.21
Reproductive NOAEL	5	99.05	66.03
Carcinogenic NOAEL	10	198.10	132.07

^1^ This value is based on an LOAEL, as effects were observed at the lowest tested dose.

**Table 2:** Selected aggregated TCPy HBM data from HBM4EU-aligned studies. For France, some values are not reported (n.r.) due to high percentage of samples below the level of detection.

Population Group	Country	P50 µg/L	P95 µg/L	Upper 95 CI µg/L
Children	Belgium	1.22	3.24	5.05
	Cyprus	6.52	13.82	15.74
	France	n.r.	n.r.	n.r.
	Israel	2.80	18.38	28.84
	Slovenia	0.61	3.08	4.92
	The Netherlands	1.13	3.49	5.55
Adults	France	n.r.	n.r	0.06
	Germany	0.82	2.87	3.87
	Iceland	0.61	2.07	3.30
	Israel	2.75	11.22	55.22
	Portugal	1.86	7.35	8.37
	Switzerland	0.97	3.64	4.72

**Table 3:** Margins of exposure ranges for the HBM4EU-aligned studies and different endpoints. P50 represents the average population, P95 the high exposed group and upper CI P95 the most exposed individuals.

Population Group	Endpoint	MoE Range P50	MoE Range P95	MoE Range Upper CI P95
Children 6–11 2014–2020	Overall LOAEL Long-term Short-term AChE Carcinogenicity	607–6462 203–2154 203–2154 47,180–215,519	215–1287 72–429 72–429 7184–42,926	137–804 46–268 46–268 4579–26,824
Adults 20–39 2014–2021	Overall LOAEL Long-term Short-term AChE Carcinogenicity	2159–7244 720–2415 720–2415 72,010–241,585	529–2870 176–957 176–957 17,648–95,701	108–1800 36–600 36–600 3587–60,030

**Table 4:** Margins of exposure for the different endpoints estimated for the studies covering the Spanish population. HBM4EU data for Portugal are included for comparison.

Study	Endpoint	MoE P50	MoE P95	MoE Max
Roca et al. [28]	Overall LOAEL	1201	320	33
Valencia	Long-term	400	107	11
Children 6–11	Short-term AChE	400	107	11
N = 125; 2010	Carcinogenicity	4007	1066	112
Fernandez et al. [29]	Overall LOAEL	3504	357	39
Valencia	Long-term	1168	119	13
Children 5–12	Short-term AChE	1168	119	13
N = 568; 2016	Carcinogenicity	116,876	11,920	1292
Suarez et al. [30]	Overall LOAEL	247,500	58,929	4091
Andalusia	Long-term	82,500	19,643	1364
Adolescents 15–17	Short-term AChE	82,500	19,643	1364
N = 117; 2017–2019	Carcinogenicity	5,282,675	1,257,780	87,317
Llop et al. [31]	Overall LOAEL	12,122	1800	51
Valencia	Long-term	4041	600	17
Pregnant women	Short-term AChE	4041	600	17
N = 573; 2003–2006	Carcinogenicity	404,186	60,030	1689
Fernandez et al. [32]	Overall LOAEL	2970	752	354
Valencia	Long-term	990	251	118
Lactating women	Short-term AChE	990	251	118
N = 116; 2015	Carcinogenicity	99,050	25,076	11,792
Gari et al., [33]	Overall LOAEL	2475		675
Catalonia	Long-term	825		225
Adults	Short-term AChE	825		225
N = 80; year not reported	Carcinogenicity	82,542		22,511
Gari et al., [33]	Overall LOAEL	1414		297
Catalonia	Long-term	471		99
Farm workers	Short-term AChE	471		99
N = 45; year not reported	Carcinogenicity	47,167		9905
HBM4EU	Overall LOAEL	3193	808	710
Portugal	Long-term	1064	269	237
Adults	Short-term AChE	1064	269	237
N = 296; 2019–2020	Carcinogenicity	106,477	26,952	23,680

**Table 5:** Graphic representation of the identified risk levels. Color code: RED: confirmed concern; ORANGE: possible concern (only applicable to OA and ST); YELLOW: concerns cannot be excluded; GREEN: risk cannot be excluded due to the concerns on genotoxicity but is expected to be very low. The letters refer to the PoD(s) reaching the reported concern level: AO—overall PoD; ST—short term AChE, LT—long term NOAEL, C—carcinogenicity, All—all PoDs at this level.

Study	Population Group	Country/Region	Average Population	High Exposed Group	Highest Individual
Roca et al. [28]	Children	ES-Valencia	C	C	All
Fernandez [29]	Children	ES-Valencia	ST	ST	All
Suarez et al. [30]	adolescents	ES-Andalusia			ST/C
HBM4EU-aligned studies	Children	*Belgium*	OA/ST	All	ST
	Children	*Cyprus*	ST	All	All
	Children	*Israel*	All	All	All
	Children	*Netherlands*	OA/ST	All	ST
	Children	*Slovenia*	OA/ST	All	ST
Llop et al. [31]	Pregnant women	ES-Valencia		All	All
Fernandez [32]	lactating women	ES-Valencia	All	OA/ST	OA/ST
Gari et al. [33]	Adults	ES-Catalonia	All	No data	OA/ST
Gari et al. [33]	farm workers	ES-Catalonia	All	No data	OA/ST
HBM4EU-aligned studies	Adults	*Germany*	OA/ST	All	All
	Adults	*Iceland*	OA/ST	All	All
	Adults	*Israel*	All	OA/ST	All
	Adults	*Portugal*	OA/LT/ST	OA/ST	OA/ST
	Adults	*Switzerland*	OA/LT/ST	All	All

## Data Availability

Summary of EFSA toxicity assessments, PRIMo model, and monitored levels in food are available through the documents published in the EFSA Journal https://efsa.onlinelibrary.wiley.com/journal/18314732, (accessed on 25 March 2022) at Open-EFSA web https://open.efsa.europa.eu/, (accessed on 25 March 2022), or Zenodo https://zenodo.org/record/6322020#.YkSAbChBzD4, (accessed on 25 March 2022).

## Supplementary Materials

The following supporting information can be downloaded at: https://www.mdpi.com/article/10.3390/toxics10060313/s1, Table S1: Individual TPCy estimations for each PRIMo diet; Table S2: Individual MOE estimations for each HBM4EU country and population group.

## References

[1] OECD (2018). Report on the OECD WORKSHOP on Sustainable Pest Management in Practice: Anticipating and Adapting to Changes in the Pesticides Regulatory Landscapes.

[2] Ganzleben C., Antignac J.-P., Barouki R., Castaño A., Fiddicke U., Klanova J., Lebret E., Olea N., Sarigiannis D., Schoeters G. (2017). Human biomonitoring as a tool to support chemicals regulation in the European Union. Int. J. Hyg. Environ. Health.

[3] Gilles L., Govarts E., Rambaud L., Vogel N., Castaño A., López M.E., Martin L.R., Koppen G., Remy S., Vrijheid M. (2021). HBM4EU combines and harmonises human biomonitoring data across the EU, building on existing capacity—The HBM4EU survey. Int. J. Hyg. Environ. Health.

[4] López M.E., Göen T., Mol H., Nübler S., Haji-Abbas-Zarrabi K., Koch H.M., Kasper-Sonnenberg M., Dvorakova D., Hajslova J., Antignac J.P. (2021). The European human biomonitoring platform-Design and implementación of a laboratory quality assurance/quality control (QA/QC) programme for selected priority chemicals. Int. J. Hyg. Environ. Health.

[5] EFSA (European Food Safety Authority) (2014). Conclusion on the peer review of the pesticide human health risk assessment of the active substance chlorpyrifos. EFSA J..

[6] EFSA (European Food Safety Authority) (2015). Reasoned opinion on the refined risk assessment regarding certain maximum residue levels (MRLs) of concern for the active substance chlorpyrifos. EFSA J..

[7] EFSA (European Food Safety Authority) (2019). Statement on the available outcomes of the human health assessment in the context of the pesticides peer review of the active substance chlorpyrifos. EFSA J..

[8] EFSA (European Food Safety Authority) (2019). Updated statement on the available outcomes of the human health assessment in the context of the pesticides peer review of the active substance chlorpyrifos-methyl. EFSA J..

[9] Bouchard M., Carrier G., Brunet R.C., Bonvalot Y., Gosselin N.H. (2005). Determination of Biological Reference Values for Chlorpyrifos Metabolites in Human Urine Using a Toxicokinetic Approach. J. Occup. Environ. Hyg..

[10] Apel P., Rousselle C., Lange R., Sissoko F., Kolossa-Gehring M., Ougier E. (2020). Human biomonitoring initiative (HBM4EU)—Strategy to derive human biomonitoring guidance values (HBM-GVs) for health risk assessment. Int. J. Hyg. Environ. Health.

[11] European Food Safety Authority (2011). Conclusion on the peer review of the pesticide risk assessment of the active substance chlorpyrifos. EFSA J..

[12] Anastassiadou M., Brancato A., Cabrera L.C., Ferreira L., Greco L., Jarrah S., Kazocina A., Leuschner R., Magrans J.O., EFSA (European Food Safety Authority) (2019). Pesticide Residue Intake Model-EFSA PRIMo revision 3.1. EFSA Support. Publ..

[13] European Food Safety Authority (2014). The 2012 European Union Report on pesticide residues in food. EFSA J..

[14] European Food Safety Authority (2015). The 2013 European Union report on pesticide residues in food. EFSA J..

[15] EFSA (European Food Safety Authority) (2016). The 2014 European Union report on pesticide residues in food. EFSA J..

[16] EFSA (European Food Safety Authority) (2017). The 2015 European Union report on pesticide residues in food. EFSA J..

[17] EFSA (European Food Safety Authority) (2018). The 2016 European Union report on pesticide residues in food. EFSA J..

[18] EFSA (European Food Safety Authority) (2019). Scientific report on the 2017 European Union report on pesticide residues in food. EFSA J..

[19] Medina-Pastor P., Triacchini G., EFSA (European Food Safety Authority) (2020). The 2018 European Union report on pesticide residues in food. EFSA J..

[20] Cabrera L.C., Pastor P.M., EFSA (European Food Safety Authority) (2021). The 2019 European Union report on pesticide residues in food. EFSA J..

[21] Nolan R., Rick D., Freshour N., Saunders J. (1984). Chlorpyrifos: Pharmacokinetics in human volunteers. Toxicol. Appl. Pharmacol..

[22] Aylward L.L., Irwin K., St-Amand A., Nong A., Hays S.M. (2018). Screening-level Biomonitoring Equivalents for tiered interpretation of urinary 3-phenoxybenzoic acid (3-PBA) in a risk assessment context. Regul. Toxicol. Pharmacol..

[23] Eaton D.L., Daroff R.B., Autrup H., Bridges J., Buffler P., Costa L.G., Coyle J., McKhann G., Mobley W.C., Nadel L. (2008). Review of the Toxicology of Chlorpyrifos With an Emphasis on Human Exposure and Neurodevelopment. Crit. Rev. Toxicol..

[24] Arnold S.M., Morriss A., Velovitch J., Juberg D., Burns C.J., Bartels M., Aggarwal M., Poet T., Hays S., Price P. (2015). Derivation of human Biomonitoring Guidance Values for chlorpyrifos using a physiologically based pharmacokinetic and pharmacodynamic model of cholinesterase inhibition. Regul. Toxicol. Pharmacol..

[25] Griffin P., Mason H., Heywood K., Cocker J. (1999). Oral and dermal absorption of chlorpyrifos: A human volunteer study. Occup. Environ. Med..

[26] Lange R., Apel P., Rousselle C., Charles S., Sissoko F., Kolossa-Gehring M., Ougier E. (2021). The European Human Biomonitoring Initiative (HBM4EU): Human biomonitoring guidance values for selected phthalates and a substitute plasticizer. Int. J. Hyg. Environ. Health.

[27] Yusà V., Fernández S.F., Dualde P., López A., Lacomba I., Coscollà C. (2022). Exposure to non-persistent pesticides in the Spanish population using biomonitoring: A review. Environ. Res..

[28] Roca M., Miralles-Marco A., Ferré J., Pérez R., Yusà V. (2014). Biomonitoring exposure assessment to contemporary pesticides in a school children population of Spain. Environ. Res..

[29] Fernández S.F., Pardo O., Corpas-Burgos F., Yusà V. (2020). Exposure and cumulative risk assessment to non-persistent pesticides in Spanish children using biomonitoring. Sci. Total Environ..

[30] Suárez B., Vela-Soria F., Castiello F., Olivas-Martinez A., Acuña-Castroviejo D., Gómez-Vida J., Olea N., Fernández M.F., Freire C. (2021). Organophosphate pesticide exposure, hormone levels, and interaction with PON1 polymorphisms in male adolescents. Sci. Total Environ..

[31] Llop S., Murcia M., Iñiguez C., Roca M., González L., Yusà V., Rebagliato M., Ballester F. (2017). Distributions and determinants of urinary biomarkers of organophosphate pesticide exposure in a prospective Spanish birth cohort study. Environ. Health.

[32] Fernández S.F., Pardo O., Adam-Cervera I., Montesinos L., Corpas-Burgos F., Roca M., Pastor A., Vento M., Cernada M., Yusà V. (2020). Biomonitoring of non-persistent pesticides in urine from lactating mothers: Exposure and risk assessment. Sci. Total Environ..

[33] Garí M., González-Quinteiro Y., Bravo N., Grimalt J.O. (2018). Analysis of metabolites of organophosphate and pyrethroid pesticides in human urine from urban and agricultural populations (Catalonia and Galicia). Sci. Total Environ..

[34] EFSA Scientific Committee (2012). Guidance on selected default values to be used by the EFSA Scientific Committee, Scientific Panels and Units in the absence of actual measured data. EFSA J..

[35] More S., Bampidis V., Benford D., Bragard C., Halldorsson T., Bennekou S.H., Koutsoumanis K., Machera K., Naegeli H., EFSA Scientific Committee (2021). Statement on the derivation of Health-Based Guidance Values (HBGVs) for regulated products that are also nutrients. EFSA J..

[36] ECHA (2012). Guidance on Information Requirements and Chemical Safety Assessment. Chapter, R.8: Characterisation of Dose [Concentration]-Response for Human Health.

[37] EFSA Scientific Committee (2012). Scientific Opinion on the applicability of the Margin of Exposure approach for the safety assessment of impurities which are both genotoxic and carcinogenic in substances added to food/feed. EFSA J..

[38] Bravo N., Peralta S., Grimalt J.O., Martínez M., Rovira J., Schuhmacher M. (2020). Organophosphate metabolite concentrations in maternal urine during pregnancy. Environ. Res..

[39] Li Y., Wang X., McKenzie J.F., Mannetje A.T., Cheng S., He C., Leathem J., Pearce N., Sunyer J., Eskenazi B. (2022). Pesticide exposure in New Zealand school-aged children: Urinary concentrations of biomarkers and assessment of determinants. Environ. Int..

[40] Dalmolin S.P., Dreon D.B., Thiesen F.V., Dallegrave E. (2020). Biomarkers of occupational exposure to pesticides: Systematic review of insecticides. Environ. Toxicol. Pharmacol..

[41] Trunnelle K.J., Bennett D.H., Tulve N.S., Clifton M.S., Davis M.D., Calafat A.M., Moran R., Tancredi D.J., Hertz-Picciotto I. (2014). Urinary pyrethroid and chlorpyrifos metabolite concentrations in Northern California families and their relationship to indoor residential insecticide levels, part of the Study of Use of Products and Exposure Related Behavior (SUPERB). Environ. Sci. Technol..

