# Supplementary material for: Improving the Risk Assessment of Pesticides through the Integration of Human Biomonitoring and Food Monitoring Data: A Case Study for Chlorpyrifos
Source: Toxics. 2022 Jun 9;10(6):313. doi: 10.3390/toxics10060313 (PMC9228629; doi:10.3390/toxics10060313)
Supplement: Supplementary file 1 [file toxics-10-00313-s001.zip › toxics-1735646-supplementary.pdf]

Improving the risk assessment of pesticides through the integration of human biomonitoring and food monitoring data. A case study for chlorpyrifos

Jose V. Tarazona [1,2], M. Carmen González-Caballero [1], Mercedes de Alba-Gonzalez [1], Susana Pedraza-Diaz [1], Ana Cañas [1], Noelia Dominguez-Morueco [1], Marta Esteban-López[1], Irene Cattaneo [2], Andromachi Katsonouri [3], Konstantinos C. Makris [4], Thorhallur I. Halldorsson [5,6], Kristin Olafsdottir [7], Jan-Paul Zock [8], Jonatan Dias [9], Annelies de Decker [10], Bert Morrens [11], Tamar Berman [12], Zohar Barnett-Itzhaki [12,13], Christian Lindh [14], Liese Gilles [15], Eva Govarts [15], Greet Schoeters [15, 16], Till Weber[17] Marike Kolossa-Gehring[17], Tiina Santonen[18] and Argelia Castaño [1,*]

<u>Supplementary material</u>

[1] National Centre for Environmental Health, Instituto de Salud Carlos III, Madrid, Spain; mcgonzalez@isciii.es; malba@isciii.es; spedraza@isciii.es; acanas@isciii.es; ndominguez@isciii.es; castano@isciii.es

[2] European Food Safety Authority (EFSA), Parma, Italy; jose.tarazona@efsa.europa.eu; irene.cattaneo@ext.efsa.europa.eu

[3] Cyprus State General Laboratory, Ministry of Health, Nicosia, Cyprus; akatsonouri@sgl.moh.gov.cy

[4] Cyprus International Institute for Environmental and Public Health, Cyprus University of Technology, Limassol, Cyprus; konstantinos.makris@cut.ac.cy

[5] Faculty of Food Science and Nutrition, School of Health Sciences, University of Iceland, Reykjavik, Iceland; tih@hi.is

[6] Department of Epidemiology Research, Statens Serum Institut, Copenhagen, Denmark

[7] Department of Pharmacology and Toxicology, University of Iceland, Reykjavik, Iceland; stinaola@hi.is

[8] National Institute for Public Health and the Environment (RIVM), Bilthoven, the Netherlands, jan-paul.zock@rivm.nl

[9] Wageningen Food Safety Research (WFSR), Wageningen, the Netherlands, jonatan.dias@wur.nl

[10] APB Provinciaal Instituut voor Hygiëne, Antwerpen, Belgium; Annelies.DEDECKER@provincieantwerpen.be

[11] Department of Sociology, University of Antwerp, Antwerpen, Belgium; bert.morrens@uantwerpen.be

[12] Ministry of Health, Israel; tamar.berman@MOH.GOV.IL

[13] Ruppin Research Group in Environmental and Social Sustainability, Ruppin Academic Center, Israel; zoharba@ruppin.ac.il

[14] Division of Occupational and Environmental Medicine, Institute of Laboratory Medicine, Lund University, Lund, Sweden; christian.lindh@med.lu.se

[15] VITO Health, Flemish Institute for Technological Research (VITO), Mol, Belgium; eva.govarts@vito.be; liese.gilles@vito.be; greet.schoeters@vito.be

[16] Department of Biomedical Sciences, University of Antwerp, Antwerp, Belgium

[17] German Environment Agency (UBA), Berlin, Germany; till.weber@uba.de; marike.kolossa@uba.de

[18] Finnish Institute of Occupational Health, Helsinki, Finland, tiina.santonen@ttl.fi

* Correspondence: castano@isciii.es

Table S1: Individual TPCy estimations for each PRIMo diet.

DIET CODE*	**YEAR** (Number corresponds to EFSA report as referenced below)							
	**2012**[1]	**2013**[2]	**2014**[3]	**2015**[4]	**2016**[5]	**2017**[6]	**2018**[7]	**2019**[8]
**DE child**	14.86	15.47	14.99	13.36	2.99	2.42	2.20	1.65
**DE general**						1.10	0.94	0.74
**DE women 14-50 yr**						1.16	1.00	0.76
**DK adult**					0.65	0.38	0.39	0.27
**DK child**	10.75	10.13	9.41	9.49	1.44	1.19	1.17	0.50
**ES adult**					1.01	1.00	0.83	0.58
**ES child**	7.21	6.84	6.55	6.12	1.58	1.41	1.24	0.81
**FI 3 yr**						0.61	0.75	0.72
**FI 6 yr**						0.50	0.57	0.58
**FI adult**					0.64	0.48	0.35	0.33
**FR adult**					1.09	0.63	0.54	0.32
**FR child 3 15 yr**						1.74	1.53	1.02
**FR infant**					0.92	0.38	0.39	0.38
**FR toddler 2 3 yr**	9.46	8.34	7.90	7.96	1.68	1.28	1.16	0.82
**GEMS/Food G06**						1.74	1.54	0.94
**GEMS/Food G07**						1.43	1.31	1.05
**GEMS/Food G08**						1.32	1.20	1.00
**GEMS/Food G10**						1.33	1.22	0.96
**GEMS/Food G11**						1.31	1.25	1.08
**GEMS/Food G15**						1.34	1.17	0.91
**IE adult**					1.97	1.18	1.16	0.90
**IE child**						0.20	0.21	0.11
**IT adult**					0.91	0.83	0.70	0.34
**IT toddler**	7.66				1.32	1.19	1.04	0.44
**LT adult**					0.39	0.35	0.34	0.39
**NL child**	13.42	12.42	12.11	11.12	2.59	1.55	1.52	1.12
**NL general**					1.13	0.82	0.73	0.63
**NL toddler**						2.35	2.43	1.72
**PL general**					0.27	0.19	0.23	0.45
**PT general**	7.88	7.46	6.95	6.39	1.25	0.93	0.93	0.81
**RO general**						1.09	0.94	0.66
**SE general**	8.11		6.09	6.43	1.27	1.10	1.14	0.90
**UK adult**				6.21	0.63	0.51	0.49	0.35
**UK infant**				10.28	1.17	0.93	0.99	0.73
**UK toddler**	7.52	6.79	6.59	6.60	1.50	1.30	1.24	0.92
**UK vegetarian**					0.81	0.66	0.62	0.44
**WHO cluster diet B**	12.58	11.35	10.24		2.34			
**WHO cluster diet D**	8.38				1.28			
**WHO cluster diet E**		6.17			1.26			
**WHO Cluster diet F**					1.18			
**WHO regional diet**					0.94			

* Codes for national diets follow official EU abbreviations. GEMS and WHO are generic diets, see EFSA references below for details.

1. European Food Safety Authority, 2014. The 2012 European Union Report on pesticide residues in food. EFSA Journal 2014; 12(12):3942, 156 pp. doi:10.2903/j.efsa.2014.3942.
2. European Food Safety Authority, 2015. The 2013 European Union report on pesticide residues in food. EFSA Journal 2015; 13(3):4038, 169 pp. doi:10.2903/j.efsa.2015.4038.
3. EFSA (European Food Safety Authority), 2016. The 2014 European Union report on pesticide residues in food. EFSA Journal 2016; 14(10):4611, 139 pp. doi: 10.2903/j.efsa.2016.4611.
4. EFSA (European Food Safety Authority), 2017. The 2015 European Union report on pesticide residues in food. EFSA Journal 2017; 15(4):4791, 134 pp. doi: 10.2903/j.efsa.2017.4791.
5. EFSA (European Food Safety Authority), 2018. The 2016 European Union report on pesticide residues in food. EFSA Journal 2018;16(7):5348, 139 pp. https://doi.org/10.2903/j.efsa.2018.5348.
6. EFSA (European Food Safety Authority), 2019. Scientific report on the 2017 European Union report on pesticide residues in food. EFSA Journal 2019;17(6):5743, 152 pp. https://doi.org/10.2903/j.efsa.2019.5743.
7. EFSA (European Food Safety Authority), Medina-Pastor, P and Triacchini, G, 2020. The 2018 European Union report on pesticide residues in food. EFSA Journal 2020;18(4):6057, 103 pp. https://doi.org/10.2903/j.efsa.2020.6057
8. EFSA (European Food Safety Authority), Carrasco Cabrera, L and Medina Pastor, P, 2021. The 2019 European Union report on pesticide residues in food. EFSA Journal 2021;19(4):6491, 89 pp. https://doi.org/10.2903/j.efsa.2021.6491.

Table S2. Individual MOE estimations for each HBM4EU country and population group.

**Population group**	**Country**	**Endpoint**	MOE P50	MOE P95	MOE UCIP95
adults	*Portugal*	*Overall LOAEC*	3192.7	808.2	710.0
adults	*Switzerland*	Overall LOAEC	6123.7	1631.4	1257.9
adults	*Israel*	*Overall LOAEC*	2159.2	529.2	**107.6**
adults	*Iceland*	*Overall LOAEC*	6387.1	2869.6	1800.0
adults	*Germany*	*Overall LOAEC*	7243,9	2069.7	1534.9
children	*Slovenia*	*Overall LOAEC*	6462.1	1287.1	804.3
children	*The Netherlands*	*Overall LOAEC*	3498.2	1135.4	712.9
children	*Belgium*	*Overall LOAEC*	3235.3	1223.1	784.8
children	*Cyprus*	*Overall LOAEC*	607.6	**286.6**	**251.5**
children	*Israel*	*Overall LOAEC*	1414.6	**215.4**	**137.3**
adults	*Portugal*	*Long-term*	*1064.2*	269.4	236.7
adults	*Switzerland*	*Long-term*	*2041.2*	543.8	419.3
adults	*Israel*	*Long-term*	719.7	176.4	**35.9**
adults	*Iceland*	*Long-term*	*2129.0*	956.5	600.0
adults	*Germany*	*Long-term*	*2414.6*	689.9	511.6
children	*Slovenia*	*Long-term*	*2154.0*	429.0	268.1
children	*The Netherlands*	*Long-term*	*1166.1*	378.5	237.6
children	*Belgium*	*Long-term*	*1078.4*	407.7	261.6
children	*Cyprus*	*Long-term*	202.5	**95.5**	**83.8**
children	*Israel*	*Long-term*	471.5	**71.8**	**45.8**
adults	*Portugal*	*Short-term AchE*	1064.2	269.4	236.7
adults	*Switzerland*	*Short-term AchE*	2041.2	543.8	419.3
adults	*Israel*	*Short-term AchE*	719.7	176.4	**35.9**
adults	*Iceland*	*Short-term AchE*	2129.0	956.5	600.0

adults	*Germany*	*Short-term AchE*	2414.6	689.9	511.6
children	*Slovenia*	*Short-term AchE*	2154.0	429.0	<u>268.1</u>
children	*The Netherlands*	*Short-term AchE*	1166.1	378.5	<u>237.6</u>
children	*Belgium*	*Short-term AchE*	1078.4	407.7	<u>261.6</u>
children	*Cyprus*	*Short-term AchE*	<u>202.5</u>	**95.5**	**83.8**
children	*Israel*	*Short-term AchE*	471.5	**71.8**	**45.8**
adults	*Portugal*	Carcinogenicity	*106476.8*	26952.4	23679.7
adults	*Switzerland*	Carcinogenicity	*204226.8*	54408.1	41949.9
adults	*Israel*	Carcinogenicity	72010.2	17647.8	**3587.4**
adults	*Iceland*	Carcinogenicity	*213010.7*	95700.5	60030.3
adults	*Germany*	Carcinogenicity	*241585.4*	69024.4	51188.6
children	*Slovenia*	Carcinogenicity	*215518.9*	42925.9	26823.9
children	*The Netherlands*	Carcinogenicity	*116669.6*	37866.3	23777.5
children	*Belgium*	Carcinogenicity	*107900.3*	40790.0	26173.7
children	*Cyprus*	Carcinogenicity	20263.9	**9557.1**	**8389.3**
children	*Israel*	Carcinogenicity	47179.7	**7184.4**	**4578.9**
